# Vertical Leakage in GaN-on-Si Stacks Investigated by a Buffer Decomposition Experiment

**DOI:** 10.3390/mi11010101

**Published:** 2020-01-17

**Authors:** Alaleh Tajalli, Matteo Borga, Matteo Meneghini, Carlo De Santi, Davide Benazzi, Sven Besendörfer, Roland Püsche, Joff Derluyn, Stefan Degroote, Marianne Germain, Riad Kabouche, Idriss Abid, Elke Meissner, Enrico Zanoni, Farid Medjdoub, Gaudenzio Meneghesso

**Affiliations:** 1 Department of Information Engineering, University of Padova, 35151 Padova, Italyborgamat@dei.unipd.it (M.B.); davide.benazzi@studenti.unipd.it (D.B.);; 2 Fraunhofer Institute for Integrated Systems and Device Technology IISB, 91058 Erlangen, Germany; Sven.Besendoerfer@iisb.fraunhofer.de (S.B.); elke.meissner@iisb.fraunhofer.de (E.M.); 3 EpiGaN, 3500 Hasselt, Belgium; Roland.Puesche@epigan.com (R.P.); joff.derluyn@epigan.com (J.D.); stefan.degroote@epigan.com (S.D.); Marianne.Germain@epigan.com (M.G.); 4 IEMN-CNRS, 59652 Villeneuve d’Ascq, France; riad.kabouche@ed.univ-lille1.fr (R.K.); idriss.abid.etu@univ-lille.fr (I.A.); farid.medjdoub@iemn.univ-lille1.fr (F.M.)

**Keywords:** Gallium nitride (GaN) high-electron-mobility transistors (HEMTs), vertical breakdown voltage, buffer trapping effect

## Abstract

We investigated the origin of vertical leakage and breakdown in GaN-on-Si epitaxial structures. In order to understand the role of the nucleation layer, AlGaN buffer, and C-doped GaN, we designed a sequential growth experiment. Specifically, we analyzed three different structures grown on silicon substrates: AlN/Si, AlGaN/AlN/Si, C:GaN/AlGaN/AlN/Si. The results demonstrate that: (i) the AlN layer grown on silicon has a breakdown field of 3.25 MV/cm, which further decreases with temperature. This value is much lower than that of highly-crystalline AlN, and the difference can be ascribed to the high density of vertical leakage paths like V-pits or threading dislocations. (ii) the AlN/Si structures show negative charge trapping, due to the injection of electrons from silicon to deep traps in AlN. (iii) adding AlGaN on top of AlN significantly reduces the defect density, thus resulting in a more uniform sample-to-sample leakage. (iv) a substantial increase in breakdown voltage is obtained only in the C:GaN/AlGaN/AlN/Si structure, that allows it to reach V_BD_ > 800 V. (v) remarkably, during a vertical I–V sweep, the C:GaN/AlGaN/AlN/Si stack shows evidence for positive charge trapping. Holes from C:GaN are trapped at the GaN/AlGaN interface, thus bringing a positive charge storage in the buffer. For the first time, the results summarized in this paper clarify the contribution of each buffer layer to vertical leakage and breakdown.

## 1. Introduction

**Figure 1:**
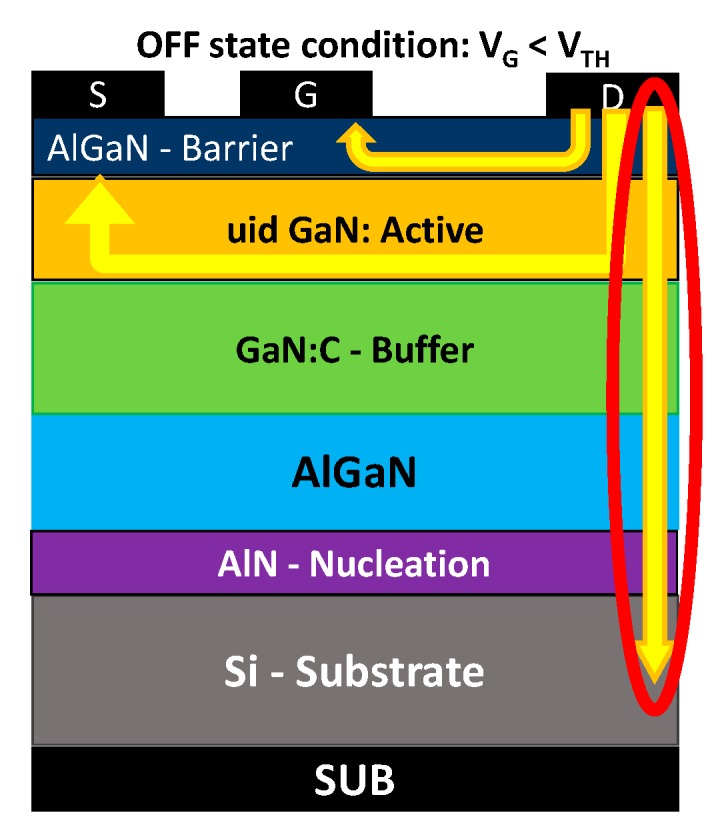
Schematic representation of GaN high-electron-mobility transistors (HEMTs), showing vertical leakage current between drain and substrate in the OFF-state condition.

Gallium nitride (GaN) high-electron-mobility transistors (HEMTs) on silicon (Si) substrate are excellent devices for power applications, and are expected to find wide application in switching power converters [1], owing to the high breakdown field of GaN (3.3 MV/cm) [2,3]. To grow GaN on Si substrates, it is necessary to develop complex buffer and transition layer architectures; these allow to obtain a good crystalline quality, which in turn leads to an improved electrical performance of the devices. However, the vertical leakage current (Figure 1) in OFF-state conditions may limit the breakdown voltage of power GaN-HEMTs. Recently, different efforts have been developed in order to improve the vertical robustness of such devices [4,5,6,7].

In this work, we investigated the vertical leakage current and breakdown of GaN-on-Si HEMTs by a sequential growth experiment. Specifically, we fabricated three tailored samples in order to analyze the contribution of the aluminum nitride (AlN) nucleation, the AlGaN buffer and the C-doped GaN layer (GaN:C) on the vertical leakage current and breakdown. Corresponding devices were characterized at room and high temperatures.

Furthermore, the physical origin of I–V hysteresis and charge trapping were analyzed; finally, UV LED irradiation was used to investigate the charge trapping phenomena in the Al-rich AlGaN buffer layers. Evidence for negative charge storage is found for the AlGaN and AlN layers, while we demonstrate that the GaN:C layer can lead to significant positive charge storage.

## 2. Experimental Details

**Figure 2:**
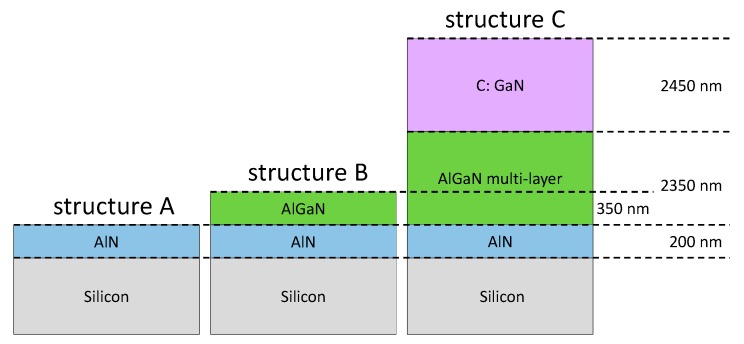
Schematic representation of the three investigated structures.

Three different structures were grown using metal organic chemical vapor deposition (MOCVD) under the same growth conditions until growth stopped (Figure 2). The first structure consisted of a 200 nm thick layer of AlN, epitaxially grown on a conductive 1 × 10^19^ cm^−3^ boron-doped silicon substrate, referred to as structure A. Structure B had an additional 350 nm thick Al_0_._70_Ga_0_._30_N layer grown on top of the AlN layer. Lastly, structure C was made of a silicon substrate, a 200 nm AlN layer, a step-graded stack of AlGaN layers with a total thickness of 2350 nm, and a 2450 nm thick carbon doped GaN layer; the carbon doping concentration was 2 × 10^19^ cm^−3^. Ohmic Ti (100 nm)/Au (400 nm) contacts were defined by photolithography; 95 × 95 μm^2^ patterns that had been isolated by N-implantation were used for electrical measurements [8]. The wafer diameter of all structures was 200 mm.

Current-voltage characterization was carried out in order to investigate leakage current, breakdown, and its physical origins at room and high temperatures as well as charge trapping before and after UV irradiation. These measurements were done with a Keysight B1505A high-voltage parameter analyzer, equipped with a high-voltage source measurement unit. The breakdown voltage was extrapolated as the voltage at which the current of 8 mA was reached. Epitaxial quality in terms of surface morphology and threading dislocation (TD) density were investigated using a JSM-7500F scanning electron microscope (SEM) (JEOL Ltd., Tokyo, Japan), a Bruker Dimension Icon atomic force microscope (AFM) in tapping mode, and a JEM-2200FS transmission electron microscope (TEM) (JEOL Ltd., Tokyo, Japan).

## 3. Results and Discussion

### 3.1. Material Quality

**Figure 3:**
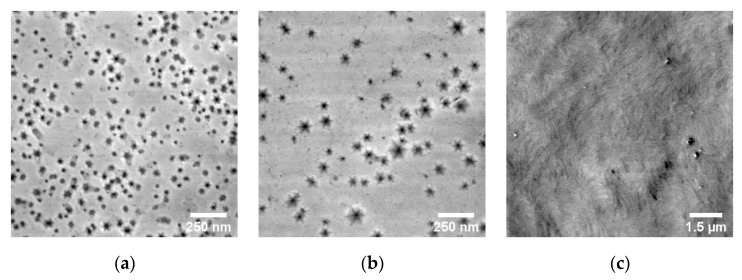
Representative scanning electron microscope (SEM) images of the as-grown surfaces of structure A (**a**), structure B (**b**), and structure C (**c**).

To investigate the material quality of the various layers, we used several experimental techniques. Using cross-sectioning and TEM-analysis, the TD-densities were estimated to be 3.2 × 10^10^ cm^−2^ for the AlN nucleation layers of all three structures, 2.3 × 10^10^ cm^−2^ for the Al_70_Ga_30_N layers of structures B and C, and 3.1 × 10^9^ cm^−2^ for the GaN:C of structure C. Surface morphologies were analyzed by SEM and AFM. The results observed by SEM are shown in Figure 3. Structure A exhibits a strongly V-pitted surface due to non-optimized nucleation conditions [9]. The V-pit density was ~1.5 × 10^10^ cm^−2^ with a typical V-pit diameter of ~60 nm. A smaller V-pit density of ~5 × 10^9^ cm^−2^, but with a larger typical V-pit diameter of ~120 nm was observed for structure B. Finally, no V-pits were observed for structure C, which was due to successful overgrowth by or below GaN:C. The corresponding rms-roughness values obtained on a scale of 5 × 5 µm^2^ by AFM are 2.35 nm, 7.55 nm, and 0.25 nm for structures A, B, and C respectively.

**Figure 4:**
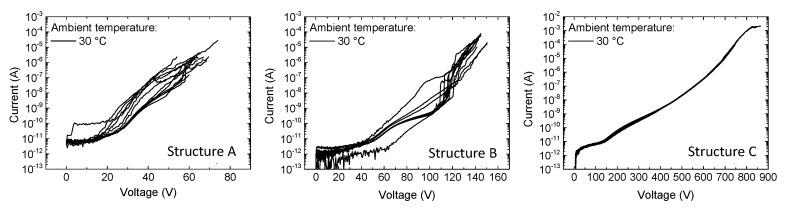
Vertical current-voltage (I–V) characteristics until breakdown for ten devices on structure A (**left**), structure B (**center**) and structure C (**right**).

A non-optimal coalescence of AlN islands on Si during the 3D growth mode gave a rough surface decorated by V-pits. Overgrowth of such a V-pitted AlN-layer led to enlarging of V-pits in subsequent layers due to a slower growth rate of V-pit side planes compared to the c-plane. Impurities, especially oxygen, can be trapped within the V-pit trace [9]. The presence of a large density of V-pits in wafers A and B resulted in a high variability in the vertical leakage characteristics, as shown in Figure 4 (notice that apart from a couple of outliers, the I–V curves of structure B were already much more uniform compared to those of structure A). On the other hand, the use of a thick GaN:C layer results in a much narrower distribution of the leakage curves due to the substantial reduction in V-pit and dislocation density obtained through the growth of a thick C-doped buffer layer.

### 3.2. Vertical Leakage

**Figure 5:**
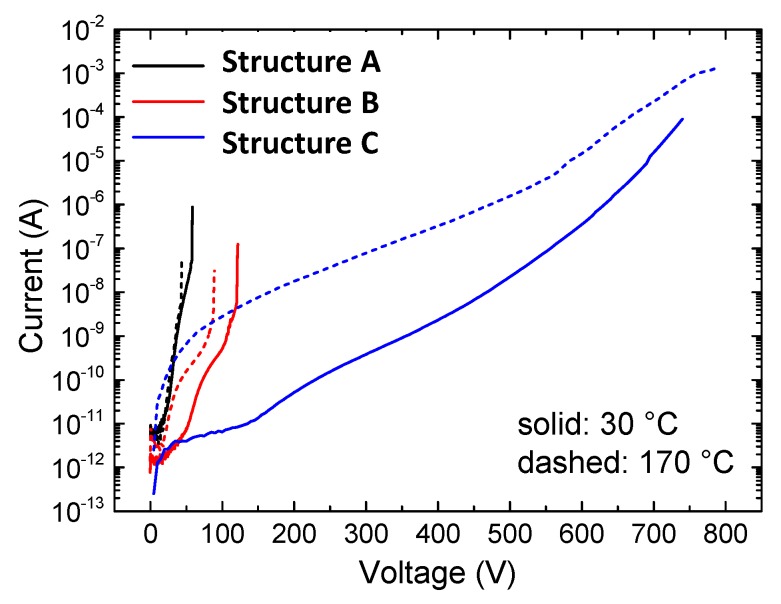
Current–voltage characteristics carried out at both low and high temperatures in the three structures under analysis.

**Figure 6:**
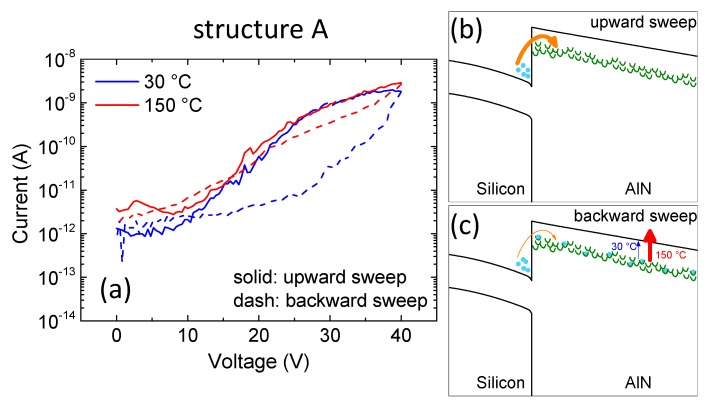
(**a**) Double sweep (upward: solid, backward: dashed) current-voltage characterization on sample A at both room and high temperature. (**b**,**c**) represent a schematic band diagram during the upward sweep and the backward sweep, respectively.

Vertical leakage characterizations with a grounded substrate and an ohmic contact sweep from 0 V up to catastrophic breakdown were performed on several samples of each structures. Figure 5 shows the typical leakage characteristics of the three heterostructures at room temperature and 170 °C. The vertical leakage current of sample A was not significantly thermally activated. This suggests that conduction through AlN is dominated by a field-driven tunneling of electrons from an electron inversion layer at the Si/AlN interface into the AlN layer (in agreement with [10]), possibly with the contribution of deep levels in AlN (Figure 6b). This process strongly depends on the availability of deep states within the AlN layer close to the interface with Si, i.e., on the local density of defects such as those described above.

**Figure 7:**
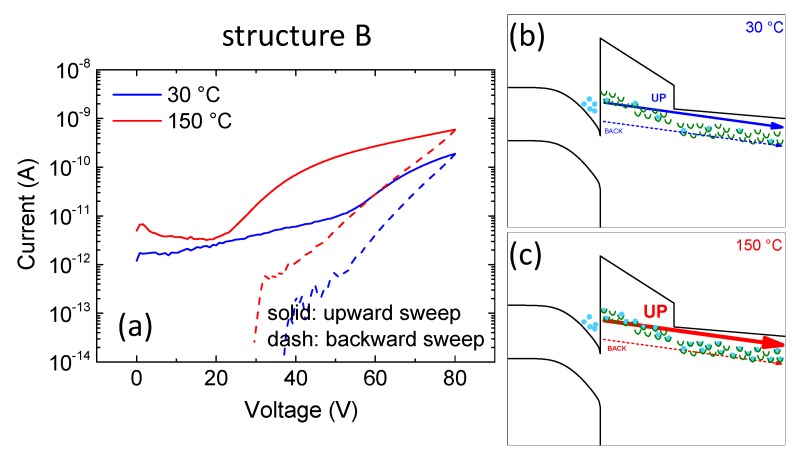
(**a**) Double sweep (upward: solid, backward: dashed) current-voltage characterization on the structure B at both room and high temperature. (**b**,**c**) represent a schematic band diagram at room and high temperature, respectively.

Contrary to structure A, the leakage current of structure B is temperature dependent. The carriers in AlGaN moved towards the top ohmic contact by means of a thermally-activated defect-assisted conduction mechanism (Figure 7b), which became the bottleneck for conduction and therefore dominated the overall behavior. This might be because the AlGaN layer showed a lower defect density as compared to AlN in terms of V-pits and threading dislocations TDs.

Blue lines in Figure 5 show the strong impact of temperature on the vertical leakage of structure C, which was compatible to the presence of a acceptor level like C_N_ within GaN:C. Thus, for this sample, conduction was likely not only related to electrons as in structures A and B, but also to holes generated by the 0.9 eV deep acceptor C_N_. Holes in GaN:C can easily flow towards the bottom of this layer and, especially at high temperature, towards the Si substrate. Hole generation was stronger at high temperatures (consistent with [3,11]).

### 3.3. Charge Trapping

In order to study the presence of charge trapping, we analyzed the hysteresis in the vertical leakage characteristics. To this end, the three structures under analysis were submitted to a double voltage sweep (upward and backward) at both room temperature and 150 °C. A large hysteresis is indicative of strong trapping effects: if negative charge is stored in the epitaxial stack during the upward sweep, the current during the backward sweep is significantly reduced [12]. The voltage step used was 1 V, while the integration time was 20 ms. Contrary to vertical leakage measurements, a maximum voltage was chosen in order to avoid catastrophic breakdown of the devices. This voltage was 40 V, 80 V, and 600 V for structures A, B, and C, respectively.

The results obtained in this study can be explained as follows:

For structure A (Figure 6a), the upward current was higher than the backward current (positive shift). This indicates that electrons that were injected from Si into AlN were trapped at defects in the AlN nucleation layer (Figure 6b). However, this effect was weaker at high temperature, leading to a considerably reduced hysteresis, possibly due to a thermally-assisted de-trapping process [13] compatible with the presence of a donor-like trap like oxygen in V-pit traces. In other words, at high temperature there was a lower amount of trapped charges during the backward sweep with respect to the room temperature condition (Figure 6b,c).

Structure B showed positive shifts for both temperatures as well. However, the shifts were much more prominent and, contrary to structure A, the shift at high temperature was stronger. As discussed above, leakage current in structure B was strongly temperature dependent due to a thermally-activated defect-assisted conduction mechanism (Figure 7b). At high temperature the contribution of trapped electrons to conduction was higher and therefore a larger hysteresis was observed.

**Figure 8:**
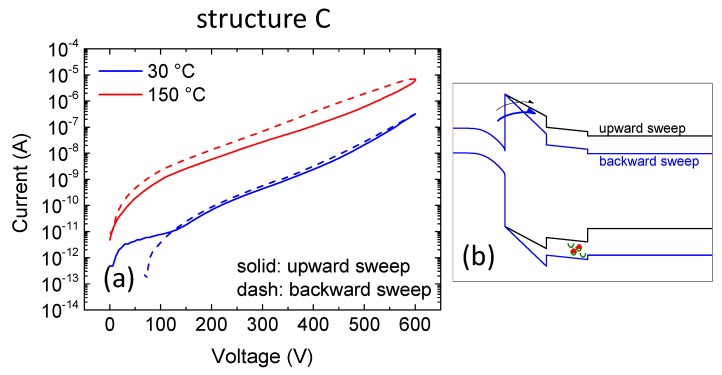
(**a**) Double sweep (upward: solid, backward: dashed) current-voltage characterization on structure C at both room and high temperature. (**b**) represents a schematic band diagram where the electrostatic effect of the positive trapped charges is highlighted.

In contrast to all other samples, sample C showed a negative shift (Figure 8a), i.e., a higher current was measured for the backward sweep, especially at high temperature. This effect was ascribed to the presence of positive charges (holes) trapped in the buffer. We suggest that holes flowed from GaN:C to the GaN:C/AlGaN heterointerface. Due to the valence band discontinuity, part of these holes may have been trapped there, thus modifying the band diagram as schematically represented in Figure 8b. This resulted in a higher current during the backward sweep. A high temperature enhanced this phenomenon, thanks to the higher number of holes available.

### 3.4. The Effect of UV Light Irradiation on Current–Voltage Charactrization

Figure 6 and Figure 7 show the presence of considerable electron trapping in the AlN and AlGaN layers. To characterize the traps which caused the experimentally observed hysteresis in the IV characteristic of structure B, we analyzed the effect of UV light with a wavelength of 385 nm on vertical leakage and charge trapping. For this, we performed I–V characterization before and after exposing the sample to UV light for 30 min.

**Figure 9:**
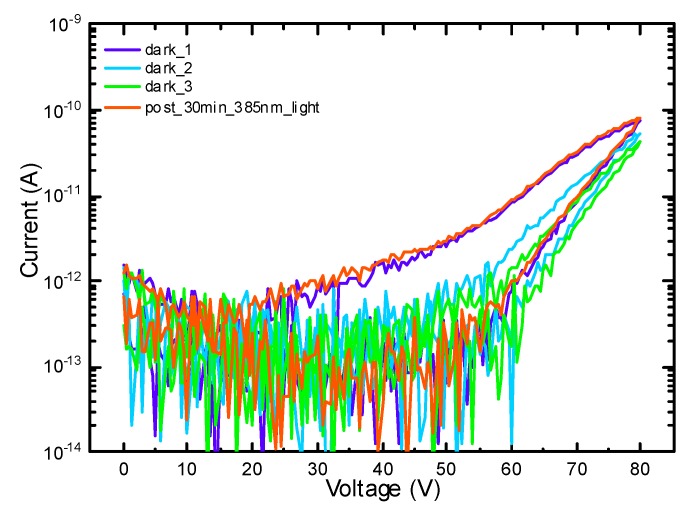
Double sweep current–voltage characterization on structure B, first, second, and third I–V on a fresh sample, then after 30 min irradiation with an LED light at a wavelength of 385 nm.

Figure 9 shows the result for the current-voltage characterization upward and backward sweep from 0 V to 80 V. Three consecutive I–V measurements were carried out on a fresh device without UV irradiation. A considerable decrease of both the current (at 80 V) and the hysteresis (ΔV at 10 pA) at the second and the third I–V characterization was observed, indicating a slow de-trapping process. Remarkably, after UV irradiation, there was a full recovery of the I–V behavior, which can be explained as follows: during the upward sweep, electrons were injected and trapped into defects of the AlN and AlGaN layers and created a substantial hysteresis. When the sample was illuminated with UV-light corresponding to E = 3.22 eV, the trapped electrons are re-emitted leading to a recovery of I–V behavior. These results confirm the existence of deep-traps, as provided by TDs especially, in the Al-rich layers and explain why the I–V curves show a slowly recovering hysteresis.

### 3.5. Vertical Breakdown

**Figure 10:**
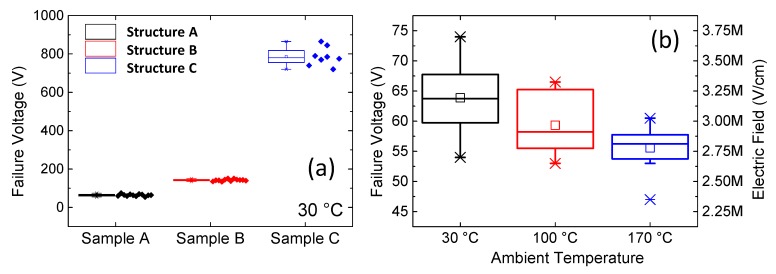
(**a**) Breakdown voltage evaluated on the three wafers under analysis; (**b**) dependence of the breakdown voltage of wafer A on temperature.

The vertical breakdown voltage of the three stacks was investigated in detail by applying a voltage sweep on the top ohmic contact while keeping the substrate grounded. Several devices were tested for each structure. The mean values obtained for samples A, B, and C were 64 V, 142 V, and 780 V, respectively (Figure 10a). When comparing the breakdown voltages of structures A and B, it is worth noticing that, by increasing the thickness of the epi-layers by 175%, the breakdown voltage increased by 120%. This indicates a non-uniform distribution of the electric field among the epi-layers. This might be ascribed to the different conductivity of the layers, as well as to the piezoelectric and spontaneous polarization charges that originated at the AlN/AlGaN interface. On the other hand, Figure 10a also demonstrates the role of the thick carbon-doped layer in increasing the vertical robustness.

For a more detailed analysis, sample A was taken in order to evaluate the temperature dependence of the critical electrical field of the AlN nucleation layer. Considering that the energy gap of AlN at 0 K is 6.15 eV [14], and that such an energy gap decreases at high temperature accordingly with the Bose–Einstein equation [14] and knowing the dependence of the breakdown field on the energy gap [15], the expected (theoretical) breakdown electric field reduction in the range of 30–170 °C was calculated to be equal to 6%. The experimental results show a reduction of the critical electric field between 30 °C and 170 °C, higher than 12% (Figure 10b).

**Figure 11:**
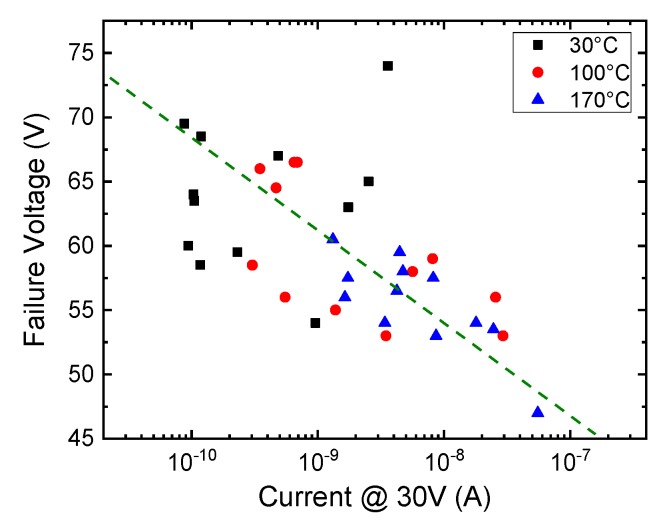
Failure voltage vs. leakage current measured at 30 V on sample A at different ambient temperatures.

To understand why the breakdown voltage has a stronger temperature dependence than predicted, we plotted the failure voltage versus the leakage current at 30 V of the devices (Figure 11). A bias of 30 V was chosen, because corresponding current is above noise level, but low enough to cause no damage to the sample. As can be noticed, a clear trend can be observed: devices with higher leakage current show a lower breakdown voltage, and consequently a lower breakdown field, of the AlN nucleation layer. This demonstrates that the breakdown process of AlN grown on Si is not only field-dependent, but also current driven. The flow of current at localized defect sites may lead to a premature breakdown of the samples, consistent with the percolation theory mentioned in Borga et al.’s research [8]. This explains why the breakdown field of AlN-on-Si (3.25 MV/cm) is much lower than that of highly crystalline AlN (up to 12 MV/cm, [8]).

## 4. Conclusions

In this work we compared three structures obtained by sequential epitaxial growth on Si substrate. We were able to separately evaluate the contribution of AlN, AlGaN, and C-doped GaN to the vertical conduction in the GaN-on-Si stack. Also, we described the related trapping processes, showing that in presence of AlN/AlGaN layers trapping is dominated by negative charge, while in the presence of C-doped GaN, positive charge trapping also plays a role.

In addition, we investigated the breakdown voltage of the samples, indicating that a C-doped GaN layer is needed to substantially increase the breakdown strength. This is ascribed to both the insulating properties of C-doped GaN, and to the increased thickness of the vertical stack. In addition, SEM and AFM analysis were used to confirm the substantial absence of extended defects like V-pits at the surface of the C-doped layer. Finally, the temperature dependence of the critical electric field of AlN grown on a Si substrate was studied. Experimental results point out that the failure of the AlN is not related to the intrinsic breakage of the semiconductor crystal, but to a defect-related phenomenon.
